# Single nuclear‐spatial transcriptomic sequencing reveals distinct puncture‐induced cell subpopulations in the intervertebral disc of a rat model

**DOI:** 10.1002/ctm2.70370

**Published:** 2025-06-13

**Authors:** Guoyan Liang, Jing Tan, Chong Chen, Yuying Liu, Yongyu Ye, Xiaolin Pan, Qiujian Zheng, Yunbing Chang, Feng‐Juan Lyu

**Affiliations:** ^1^ Department of Spine Surgery Guangdong Provincial People's Hospital (Guangdong Academy of Medical Sciences) Southern Medical University Guangzhou P.R. China; ^2^ School of Medicine Joint Center for Regenerative Medicine Research of South China University of Technology and the University of Western Australia South China University of Technology Guangzhou P.R. China; ^3^ Department of Trauma Surgery & Joint Surgery Guangdong Provincial People's Hospital (Guangdong Academy of Medical Sciences) Southern Medical University Guangzhou P.R. China; ^4^ School of Medicine South China University of Technology Guangzhou P.R. China

**Keywords:** intervertebral disc degeneration, LCN2, oxidative stress, PDGFRA, single‐nucleus RNA sequencing, spatial transcriptomic

## Abstract

**Objective:**

We aim to investigate the spatiotemporal dynamics of intervertebral disc (IVD) cell subpopulations in IVD degeneration (IVDD).

**Methods:**

To gain combined spatial and transcriptomic insights into IVDD, we employed both spatial transcriptomic sequencing (stRNA‐seq) and single nucleus RNA sequencing (snRNA‐seq) in a rat puncture‐induced IVDD model. The findings were verified in rat and human IVD by immunostaining and qRT‐PCR. Tamoxifen‐administered Pdgfra^CreERT2^;R26^tdTomato^ mice were adopted to track platelet‐derived growth factor receptor alpha (Pdgfra) positive cells.

**Results:**

Puncture response regions were revealed on day 1 post‐puncture, for which oxidative stress emerged as a prominent pathway in a Stress Zone consisting of lipocalin‐2 (Lcn2)+ annulus fibrosus (AF) cells (AFC), which propagated and migrated into nucleus pulposus (NP), playing a key role in delivering injury signals and triggering pathological processes, including ferroptosis, fibrosis, and immune reactions. In the NP, Collagen 3‐high (Col3hi) NP cells (NPC) were another induced population demonstrating a fibrochondrocyte‐like phenotype and high epithelial–mesenchymal transition activation, an important pathway involved in tissue fibrosis. Crucially, lineage tracing experiments in Pdgfra^CreERT2^;R26^tdTomat^ mice revealed the significant migration and proliferation of Pdgfra+ AFCs from the AF into the NP following puncture. These findings provide direct evidence that both Pdgfra+ AFCs and Col3hi NP cells may contribute to NP fibrosis.

**Conclusion:**

Puncture‐induced oxidative stress in a stress zone is the primary reaction playing an important role in initiating IVDD. Several puncture‐induced cell subpopulations were identified, including Lcn2+ AFC, Col3hi NPC, and Pdgfra+ AFC. Lcn2+ AFC plays a pivotal role in connecting oxidative stress with other pathological processes. Our results clarified the dual origin of Pdgfra+ cells, highlighting the contribution of AF‐derived cells to the NP during degeneration and emphasizing the complexity of cellular changes underlying NP fibrosis. Further investigation into the specific contributions of Pdgfra+ cells from different origins to fibrosis is warranted.

**Key points:**

- Puncture induced oxidative stress in a Stress Zone is the primary reaction in initiating IVDD.
- Puncture induced several IVD cell subpopulations, including Lcn2+ AFC, Col3hi NPC and Pdgfra+ AFC.
- Lcn2+ AFC plays a pivotal role in connecting oxidative stress with other pathological processes.
- Pdgfra+ cells in the NP derived from both Pdgfra+ AFC and Col3hi NPC, highlighting dual origin of NP fibrosis.

## Graphical abstract

Summary of key events initiated from Stress Zone and related cell populations in puncture‐induced IVD degeneration.

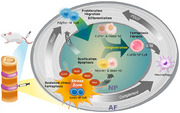

## 1 INTRODUCTION

Intervertebral disc (IVD) degeneration (IVDD) is a prevalent pathological condition that contributes to low back and neck pain, resulting in significant disability.^1^, ^2^ Affecting over 70% of individuals under 50 years and 90% of those over 50 years, IVDD imposes a substantial burden on productivity and healthcare systems.^1^ However, current therapeutic approaches demonstrate limited efficacy in ameliorating IVDD.^2^ Previously, oxidative stress and ferroptosis have been indicated to be upregulated in IVDD.^3^ Oxidative stress, resulting from an imbalance between reactive oxygen species and antioxidants, damages disc cells and the extracellular matrix.^4^ Ferroptosis, a distinct form of iron‐dependent cell death characterized by lipid peroxidation, contributes to the loss of both NP and AF cells.^5^ Targeting oxidative stress or ferroptosis could alleviate IVDD.^6^ The infiltration of inflammatory mediators^7^, ^8^, ^9^, ^10^ and decrease in extracellular matrix (ECM) and cell number^11^ have been reported in IVDD. These lead to dehydration and shrinkage of the nucleus pulposus (NP), as well as a reduction in disc height, while sustained inflammation eventually results in NP fibrosis.^12^, ^13^, ^14^, ^15^ Although some potential molecular indicators of IVDD have been reported,^16^, ^17^, ^18^ a clear view of the cellular process initiating these changes is lacking. Furthermore, the subpopulations of the IVD, as well as their temporal and spatial changes during degeneration, remain unclear. Specifically, cellular events involved in pathological processes such as cell death, fibrosis, and inflammation are poorly understood. Such information is essential for elucidating the transition from initiation to degeneration and for developing targeted therapeutic interventions.

While single‐cell RNA sequencing (scRNA‐seq) has been widely used to study IVDD, enzymatic dissociation of tissues can introduce artifacts in gene expression.^19^ Single‐nucleus RNA sequencing (snRNA‐seq) offers an alternative approach by analyzing nuclei instead of intact cells, thereby enabling single‐cell transcriptome profiling.^20^ Importantly, snRNA‐seq allows the analysis of frozen tissues, preserving the in vivo transcriptional state.^21^ However, neither technique provides information about the spatial expression within the tissue. Previous reports about scRNA‐seq in IVD were all limited with no information on the spatial context in the IVD. Spatial transcriptomic RNA sequencing (stRNA‐seq) overcomes this limitation by mapping gene expression within the intact tissue architecture.^22^ Therefore, integrating single‐cell information with the spatial context is crucial for understanding the spatiotemporal dynamics of cell subpopulations during IVDD.

Annular injury, such as tear or rupture, is a common cause of disc herniation and may represent a trigger for IVDD.^23^, ^24^ IVD puncture is a common way to establish a model to mimic injury‐induced IVDD because of its reproducibility and capacity to rapidly induce degenerative changes.^25^, ^26^, ^27^ By creating a controlled injury that resembles clinical annular tears, this model enables the study of subsequent cellular and molecular responses following IVD injury. In this study, we combined stRNA‐seq and snRNA‐seq in puncture‐induced degenerated rat IVDs to comprehensively explore the cellular progression from injury to early degeneration, reveal the spatial‐molecular architecture of IVD degeneration, and determine the contributions of various cell populations.

## 2 MATERIALS AND METHODS

### 2.1 Rat IVD degeneration model

**FIGURE 1:**
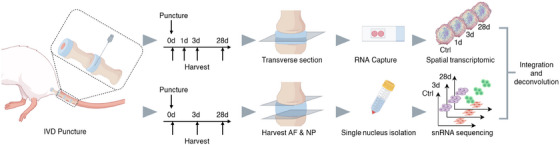
Study design workflow. Flowchart illustrating the experimental procedures, including sample processing, and the integration of stRNA‐seq and snRNA‐seq. stRNA‐seq: Spatial transcriptomic sequencing; snRNA‐seq: single nucleus RNA sequencing.

Our study aimed to specifically investigate both the early and late stages of IVD degeneration following puncture. Previous studies have demonstrated the onset of changes in IVD degeneration scores as early as 3 days post‐puncture, reflecting an acute cellular response to the induced injury.^28^, ^29^ This early time point is crucial for capturing the initial cellular and molecular events that trigger the degenerative cascade. By 28 days, the degenerative processes typically progress to a more advanced and stable, chronic state.^25^, ^30^ Therefore, we employed a rat IVD puncture model with time points at pre‐puncture, day 3, and day 28 post‐puncture for both stRNA‐seq and snRNA‐seq (Figure 1). An additional day 1 time point was included for stRNA‐seq to capture the immediate spatial changes in gene expression occurring immediately after injury. Twenty‐one healthy female Sprague–Dawley (SD) rats, aged 6–8 weeks and weighing 200–230 g, were randomly assigned to one of three experimental groups (*n* = 7 per group): control (unpunctured) group, Day 3 post‐puncture group, and Day 28 post‐puncture group. Since healthy IVDs maintain homeostasis through balanced matrix turnover by a stable cell population,^31^ we used unpunctured IVD to represent the transcriptomic status of a normal IVD over the course of a month.

The IVD puncture model was generated as previously described.^12^ After anesthetization, the coccygeal (Co) IVDs at levels Co4/5, Co5/6, Co6/7, and Co7/8 were exposed through an incision. In the control group, IVDs were collected on the same day after a sham surgery on the tail (without puncture). In the puncture groups, a 21G needle was inserted parallel to half the depth of the NP through a unilateral AF puncture, held in place for 30 s, and then withdrawn. Injury to the endplate or full penetration was carefully avoided. IVDs were collected at pre‐puncture and 1, 3, and 28 days post‐puncture for further analysis.

### 2.2 Spatial transcriptomic sequencing

stRNA‐seq was performed using the 10x Genomics Visium platform. We employed transverse sectioning through the puncture plane of the IVD to avoid decalcification of bony tissue, which may compromise RNA integrity and reduce sequencing efficiency^32^ (Figure 1). We prepared four discs per group: two for preliminary experiments that optimize permeabilization conditions and two for final spatial transcriptomic sequencing.

The Co4/5 IVDs from each group (control, Day 1, Day 3, and Day 28) were freshly isolated. The discs were transversely embedded in optimal cutting temperature compound (OCT) media on dry ice, cut into 10 µm sections using a freezing microtome (Leica CM1950), and attached (*n* = 2 per time‐point) to the 10X Genomics Visium Spatial microarray slide. The 10X Genomics Visium Spatial transcriptomics system (1st Version) has a standard capture area of 6.5 mm × 6.5 mm, with 4992 spots per capture area, providing an average resolution of 1 to 10 cells per spot. Sections were fixed and stained with H&E for digital imaging, then permeabilized and treated with a reverse transcription mix according to the instructions. The bound cDNA was subsequently released from the slide and used to prepare a library for sequencing according to the 10X Genomics Visium Spatial protocols. The libraries were sequenced using the DNBSEQ‐T7 platform (MGI Tech). Detailed protocols for data processing were provided in the Supporting Information Methods.

### 2.3 Single‐nucleus RNA sequencing

snRNA‐seq was performed exclusively on AF and NP tissues to maintain consistency across our multiomics dataset and allow direct comparison (Figure 1). Control group IVDs, as well as Day 3 and Day 28 post‐punctured IVDs, were used for snRNA‐seq. The Co4/5 and Co5/6 IVDs from three rats in each group were pooled for each time point to ensure high‐quality data with sufficient cell numbers for robust analysis. The collected IVDs were snap‐frozen and embedded in OCT, and the upper and lower cartilage endplates (CEP) were meticulously removed using a microtome at −20°C. Upon removal of the OCT, the AF and NP tissues were homogenized in ice‐cold homogenization buffer containing .25 M sucrose, 5 × 10^−3^ M CaCl_2_, 3 × 10^−3^ M MgAc_2_, 10 × 10^−3^ M Tris‐HCl (pH 8.0), .1 × 10^−3^ M EDTA, 1x protease inhibitor, and 1 U/µL RiboLock RNase inhibitor using pestle strokes. Subsequently, the homogenates were filtered through a 70 × 10^−6^ m cell strainer to collect the nuclear fraction. The nuclear fraction was mixed with an equal volume of 50% iodixanol and added on top of a 30% iodixanol solution. This solution was then centrifuged for 20 min at 10 000×*g* at 4°C and the nuclei were collected. The nuclei were resuspended in nuclear wash buffer and resuspension buffer (.04% bovine serum albumin, .2 U/µL RiboLock RNase inhibitor, 500 × 10^−3^ M mannitol, and .1 × 10^−3^ M Phenylmethanesulfonyl fluoride as a protease inhibitor in PBS) and pelleted for 5 min at 500×*g* and 4°C. Following this, the nuclei were filtered through a 40 × 10^−6^ m cell strainer to remove cell debris and large clumps. The nuclear concentration was manually assessed using trypan blue counterstaining and a hemocytometer. Finally, the nuclear concentration was adjusted to 700–1200 nuclei/µL. The suspension was loaded onto a 10X Genomics GemCode Single‐cell instrument to generate single nuclear Gel Beads‐In‐Emulsions. Library construction and sequencing were then performed following the 10X Genomics Chromium protocols. The libraries were sequenced using the Novaseq 6000 platform. Detailed protocols for data processing were provided in the Supporting Information Methods.

### 2.4 Data processing

In the spatial transcriptomic sequencing experiments, the sequencing depth (mean reads per spot) for the control, Days 1, 3, and 28 samples are 87 937, 119 734, 121 020, and 134 994, respectively, indicating good sensitivity in capturing the transcriptome. In the snRNA‐seq experiments, the estimated number of cells (nuclei) for the control, Days 3 and 28 samples are 12 431, 13 180, and 18 844, respectively, indicating successful nuclei isolation and library preparation. The sequencing depth (mean reads per cell) for the control, Days 3 and 28 samples are 32 791, 27 587, and 21 394, respectively, providing sufficient coverage for gene expression analysis. The principal component analysis (PCA) plot (Figure S1) suggested a high degree of similarity across the three groups, indicative of high‐quality sequencing data. The data were processed using Seurat R packages (Version 4.0).^33^ SPOTlight R package was utilized to determine the cell‐type composition of each spot in the spatial transcriptomic data.^34^ Differential expression genes (DEGs) analyses were performed using Seurat. Marker genes were chosen based on two criteria: (1) they ranked within the top 10 of the DEG analysis (with a threshold of log2 fold change >1.5 and a *p*‐value < .01), and (2) they have known biological significance. Cell annotation was performed in two stages: First, initial cell types were identified based on established marker genes and spatial location; Second, subclusters within each identified cell type were identified using DEGs distinct from the marker genes used in the initial identification. Where unique marker gene expression was absent, spatial distribution information was used to define cell subclusters. Gene Ontology (GO), Gene Set Enrichment Analysis (GSEA), and Gene Set Variation Analysis (GSVA) pathway enrichment analyses were conducted.^35^ Cell differentiation trajectory analysis was conducted using the Monocle3 and CytoTRACE packages.^36^, ^37^ The CellChat R package was employed to analyze the communication network among distinct cell clusters.^38^

### 2.5 Lineage tracing of Pdgfra+ descendant cells

Pdgfra+ lineage tracing was performed using *Pdgfra^CreERT2^;R26^tdTomato^* mice in an IVD puncture model with time points at control, Days 14 and 28. tdTomato expression was used to identify Pdgfra+ descendant cells. To achieve this, Pdgfra^CreERT2^ mice were crossbred with R26^tdTomato^ mice to generate Pdgfra^CreERT2^;R26^tdTomato^ mice. The CreER/loxP recombination was induced by daily intraperitoneal injections of tamoxifen (75 mg per kg body weight) for three consecutive days. After 7 days, the tail IVDs were punctured at Co6/7 and Co8/9 using a 31‐gauge needle. The puncture was guided by X‐ray rather than using a skin incision to minimize potential disturbance to the Pdgfra+ AF cells caused by open surgery. Control intact IVDs were collected from the Co7/8 and Co9/10 levels. IVD samples were harvested on Days 0, 14, and 28 after puncture. Pdgfra+ lineage cells were identified and visualized using a fluorescence microscope based on their expression of tdTomato.

### 2.6 Oxidative stress induction

A Rat IVD ex vivo organ culture model was used for oxidative stress induction. Briefly, the IVDs were isolated and randomly assigned to either the control group or the stressed group. They were cultured in DMEM containing 15% fetal bovine serum and 1% penicillin/streptomycin at 37°C in a humidified atmosphere, consistent with previous research.^39^ For the stressed group, oxidative stress was induced by adding 200 µM hydrogen peroxide (H₂O₂) to the culture medium.^40^ The medium was changed daily, and tissues were harvested after three days of culture. Following paraffin embedding and sectioning, LCN2 expression was detected by histochemical staining. The percentage of LCN2+ AF cells was then quantified and compared between the two groups.

### 2.7 Immunostaining and qRT‐PCR

Healthy and degenerative rat IVDs were collected at 1, 3, and 28 days post‐puncture. Mouse IVDs for Pdgfra+ cell lineage tracing were collected at 0, 14, and 28 days post‐puncture. The animal IVDs were fixed and decalcified as previously published.^12^ All animal IVD samples were embedded in OCT media and cut into 10 µm frozen sections.

Human IVD samples (Pfirrmann grades II–V) were obtained from 57 patients undergoing discectomy for lumbar disc herniation or spine trauma at Guangdong Provincial People's Hospital between 2020 and 2022. NP tissues were carefully isolated with a scalpel, based on the differential texture and color of NP as compared with AF and EP. The NP tissues were embedded in paraffin, sectioned at 10 µm, and processed through a standard deparaffinization and rehydration protocol using xylene and ethanol. A .4% pepsin solution (Sigma‐Aldrich) was applied and antigen retrieval was performed at 37°C for 20 min. The IVD degeneration grade of the patients was assessed using the Pfirrmann classification system.^41^ Histopathological scoring analysis was conducted using a scoring system specifically designed for rat IVD models.^42^

Safranin O/Fast Green staining for rat IVDs (Ctrl, day 1, 3, and 28) were performed according to the manufacturer's instructions (Solarbio). Immunohistochemical analysis for rodent and human IVD samples was referred to previous studies characterizing protein expression patterns within the IVDs.^8^ We used primary antibodies against lipocalin‐2 (LCN2, 1:100, Abclonal, Cat# A24538), metallothionein 2A (MT2A, 1:100, Abclonal, Cat# A2018), superoxide dismutase 2 (SOD2, 1:100, Abclonal, Cat# A21805), and Ki67 (1:100, ABclonal, Cat# A20018) in both rat and human IVDs. IVD sections were initially incubated in blocking solution (5% goat serum) for 1 h at room temperature to prevent nonspecific epitope binding. Following this, the sections were incubated overnight at 4°C with primary antibodies diluted in a blocking solution. After thorough washing, the sections were incubated with secondary antibodies (ABclonal, Cat# AS014 and AS053) at room temperature for 1 h. For immunofluorescent staining, the slides were sealed with an antifade mounting medium containing DAPI (Solarbio, 1:1000, Cat# S2110) and examined under a Leica microscope (DM2500). For immunohistochemistry staining, the tissues were processed using the diaminobenzidine kit (DAB, ZLI‐9019, ZSGB‐BIO). Finally, the human IVD tissues were counterstained with hematoxylin and photographed under the microscope.

The qRT‐PCR experiments were conducted following this procedure. AF tissues from rats or mice IVDs were meticulously isolated under a stereo microscope. Total cellular RNA was extracted using an RNA purification kit (B0004D, EZBioscience), following the manufacturer's instructions. The extracted RNA was then reverse transcribed into cDNA using the Cell to cDNA Kit PLUS II (B0003C, EZBioscience). Real‐time qPCR was executed utilizing the cDNA, 2× Color SYBR Green qPCR Master Mix (A0012‐R2, EZBioscience), and StepOnePlus PCR system (Bio‐Rad). The details of the primers are listed as follows (5′‐3′): Rat *Pdgfra*: F‐TTTTTCCTCCGGGCTATCGG/R‐GATGAGGCTCGGCCCTGT; Mouse *Pdgfra*: F‐ATGACTCCGAGGGTCTGACTT/R‐GTCCCGGTGGACACAATTTTTCG.

### 2.8 Statistical analysis

The data were presented as mean ± SEM and statistical analysis was conducted using GraphPad Prism version 9.0. The unpaired or paired Student's *t*‐tests were used for comparisons between the two groups. A one‐way ANOVA, followed by Tukey's post hoc test, was used for comparisons among multiple groups. A *p*‐value < .05 was considered statistically significant.

## 3 RESULTS

### 3.1 Integration of snRNA‐seq and stRNA‐seq identified distinct cell subpopulations

Histopathological analysis confirmed successful progressive IVDD induced by puncture over a period of 28 days (Figure S2). The cellular composition of IVDs was investigated using snRNA‐seq on days 0 (control), 3, and 28 post‐puncture. Previous studies have shown that snRNA‐seq and scRNA‐seq have comparable performance in identifying cell subsets and capturing gene expression profiles.^43^, ^44^, ^45^, ^46^, ^47^ Moreover, snRNA‐seq has been reported to excel at capturing changes in cell state.^43^, ^47^, ^48^ To validate our control snRNA‐seq data and assess potential methodological differences, we compared them with publicly available scRNA‐seq data from normal rat IVDs (GSE154884)^49^ (Figure S3).

Both methods identified key cell populations, including NP, AF, and immune cells. Correlation analysis identified DEGs with good consistency, but stress response‐related genes were higher detected in scRNA‐seq (Figure S3). We postulate that this discrepancy may be attributed to the exclusion of mitochondria in the preparation of the snRNA‐seq procedure. Furthermore, the stress induced by the enzymatic digestion used in scRNA‐seq to isolate live cells^50^, ^51^ may contribute to increased stress‐responsive gene expression, whereas snRNA‐seq uses frozen samples without enzymatic digestion. Importantly, the expression of these genes in normal discs was negligible in snRNA‐seq, consistent with our stRNA‐seq results. This finding underscores the strong concordance between snRNA‐seq and stRNA‐seq data.^43^, ^46^, ^52^

**FIGURE 2:**
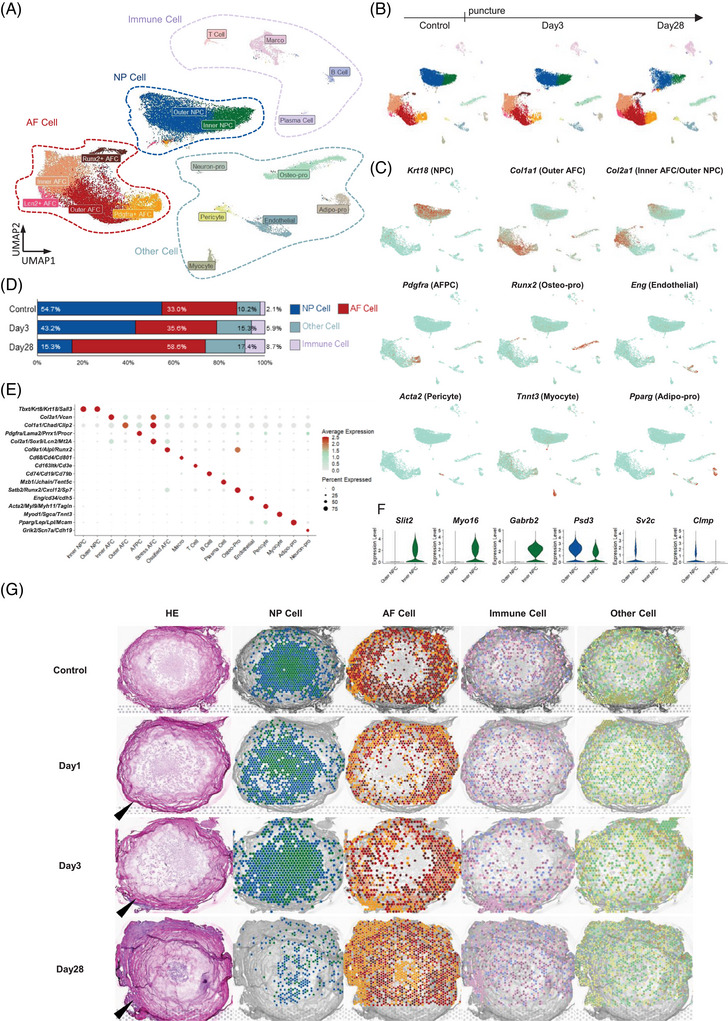
Classification and location of IVD cell subclusters using stRNA‐seq and snRNA‐seq data. (A) UMAP visualization of integrated rat IVD snRNA‐seq data from all time points, demonstrating the identification of 17 distinct cell clusters through unsupervised clustering. (B) Changes in the UMAP visualization of snRNA‐seq data following puncture. (C) Expression levels of representative marker genes in major cell clusters. (D) Changes in the proportions of the four main cell categories (NPCs, AFCs, immune cells, and other cells) following puncture. (E) Expression levels of specific marker genes in each cell cluster. (F) Differential expression levels between the outer and inner NPC clusters. (G) Relative spatial distribution of the subclusters within each main cell category at four time points. Arrowheads indicate the direction of the puncture. IVD, intervertebral disc; stRNA‐seq, spatial transcriptomic sequencing; snRNA‐seq, single nucleus RNA sequencing; NPC, nucleus pulposus cells; AFCs, annulus fibrosus cells.

The integration of snRNA‐seq and stRNA‐seq data at all time points resulted in the identification of 17 distinct cell clusters (Figure 2A,B), which were categorized into four groups: AF cells (AFCs), NP cells (NPCs), immune cells, and other cells. NPCs were the only category that continuously decreased in proportion over degeneration (Figure 2D). Figure 2C shows the location of these marker genes in the entire disc, while the marker genes of each cell subset are illustrated in Figure 2E. Both inner and outer NPCs, which were named according to their spatial location, expressed NP marker genes such as *Tbxt*, *Krt8*, and *Krt18* (Figure 2G). In addition, Inner NPCs highly expressed *Slit2*, *Myo16*, and *Gabrb2*, whereas the Outer NPCs highly expressed *Psd3*, *Sv2c*, and *Clmp* (Figure 2F). When mapping these cell subsets onto the discs, we noticed that the boundary between the inner and outer NP had disappeared, while AFCs had migrated into the inner NP by Day 28 (Figure 2G). In addition, immune cells, which originally resided outside the NP, exhibited increased presence within the Day 28 NP.

### 3.2 Analysis of AFC subsets highlighted Lcn2+ AFCs in response to puncture

**FIGURE 3:**
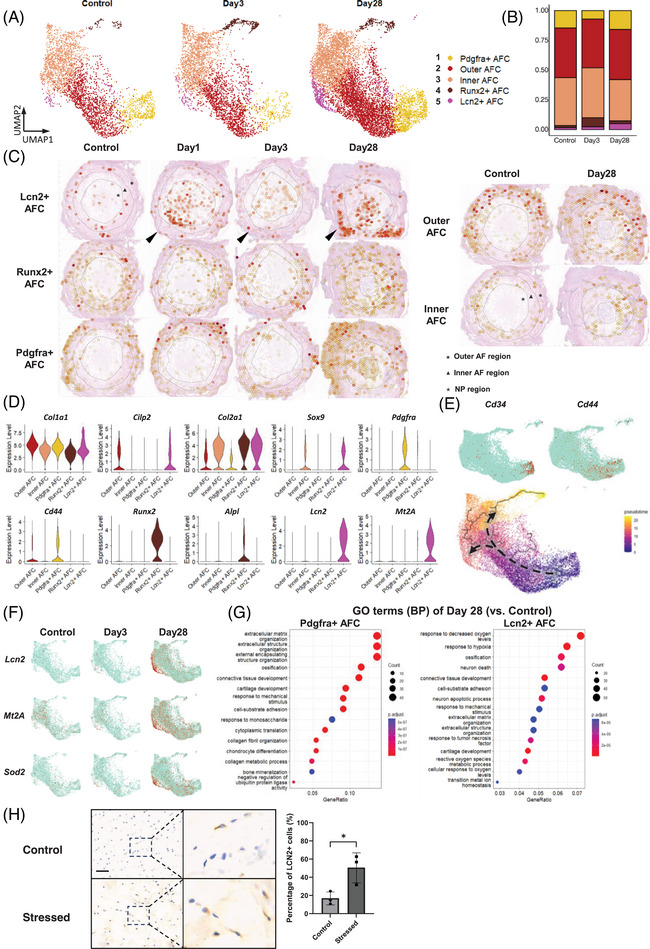
Analysis of AF cell subclusters. (A) UMAP visualization of the identification of five distinct subclusters within the AFC population. (B) Proportional representation of the five AFC subclusters. (C) Spatial distribution of the five AFC subclusters following puncture. Arrowheads indicate the direction of the puncture. The two dashed lines within each IVD delineate the boundaries between the outer and inner AF and NP regions. (D) Relative expression levels of marker genes specific to each of the five AFC subclusters. (E) Expression patterns of CD34 and CD44 indicating the root of developmental analysis. Pseudotime analysis with Monocle was used to assess cell trajectories along inferred developmental paths. (F) Distribution and expression patterns of the oxidative stress markers *Lcn2*, *Mt2A*, and *Sod2* within the AFC cluster at different time points. (G) Biological process terms obtained from GO analysis comparing changes observed in day 28 samples to control samples within the Pdgfra+AFC and Lcn2+AFC subclusters. (H) Immunochemistry staining of ex vivo IVD cultures revealed an increased percentage of Lcn2+ AFC under a hydrogen peroxide‐induced oxidative stress environment. *N* = 3 per group. Scale bar = 50 µm. *p < .05 vs. control by Student's *t*‐test was considered statistically significant. Error bars represent 95% confidence intervals (CI). AF, annulus fibrosus; AFC, AF cells; NP, nucleus pulposus; IVD, intervertebral disc; LCN2, lipocalin‐2; MT2A, metallothionein‐2; SOD2, superoxide dismutase 2.

The five AFC subsets were analyzed in greater detail (Figure 3A,B). The spatial distribution and distinctive markers of each AFC subset are shown in Figure 3C,D. Inner AFCs highly expressed *Col2a1*, whereas Outer AFCs highly expressed *Col1a1* and *Cilp2*. Runx2+ AFCs were characterized by high *Runx2* and *Alpl* levels, indicating their association with ossification during IVDD. Figure 3C shows an increase in Outer AFCs within the inner AF by day 28, concurrent with an elevated presence of Lcn2+ AFCs and Pdgfra+ AFCs in the NP.

Lcn2+ AFCs were the only AF subset that merely existed in the healthy AF. Post‐puncture, Lcn2+ AFCs continuously increased (Figure 3B), consistent with the stRNA‐seq result (Figure 3C). Importantly, only Lcn2+ AFCs exhibited upregulation of oxidative stress markers, such as *Lcn2* and *Mt2A* (Figure 3D,F). LCN2 (lipocalin‐2) is widely recognized as a biomarker of oxidative stress,^53^, ^54^, ^55^ MT2A (metallothionein‐2) acts as an antioxidant that eliminates free radicals.^56^ Further GO analysis revealed a strong response to reactive oxygen species in Lcn2+ AFCs (Figure 3G). We further tested whether oxidative stress contributed to the expansion of Lcn2+ AFCs using an ex vivo IVD organ culture system. After 3 days of culture with oxidative stress induced by hydrogen peroxide, the percentage of Lcn2+ AFCs in the Stressed group was significantly increased compared with the control group (Figure 3H). These results provide evidence that Lcn2+ AFCs are among the primary effector cells in response to oxidative stress.

An increase in Pdgfra+ AFCs was also observed following puncture (Figure 3B,C). Previous studies have suggested the progenitor potential of Pdgfra‐expressing cells during alveologenesis.^57^ In our study, Pdgfra+ AFCs highly expressed the stemness markers *CD34* and *CD44* in addition to *Pdgfra* (Figure 3D,E), suggesting the progenitor potential of Pdgfra+ AFCs. Consistently, pseudotime analysis (Figure 3E) revealed Pdgfra+ AFCs as the starting point of differentiation within the AFC clusters, with two distinct endpoints, represented by Runx2+ AFCs and Lcn2+ AFCs. GO analysis further revealed that Pdgfra+ AFCs were primarily associated with ECM organization and chondrocyte differentiation (Figure 3G).

### 3.3 Lineage tracing of Pdgfra+ AFCs clarified a portion of AF‐derived Pdgfra+ NPCs

**FIGURE 4:**
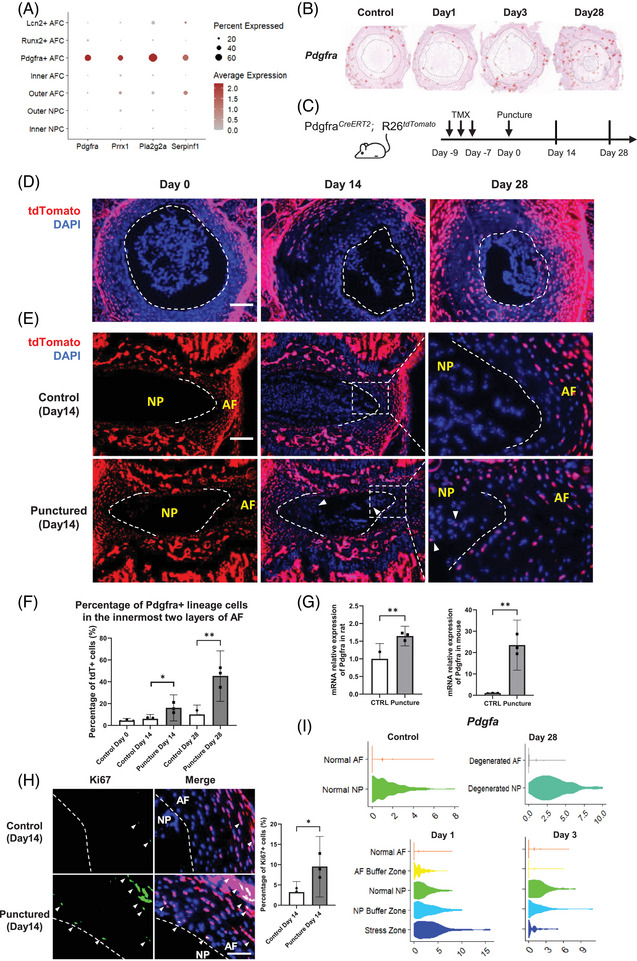
Distribution of Pdgfra+ descendant cells in the mouse IVD puncture model. (A) Expression levels of *Pdgfra, Prrx1, Pla2g2a*, and *Serpinf1* genes in AFCs and NPCs. (B) Spatiotemporal distribution of *Pdgfra* in stRNA‐seq data. The two dashed lines within each IVD delineate the boundaries between the outer and inner AF and NP regions. (C) Schematic illustrating the lineage tracing procedure used to track *Pdgfra*+ descendant cells. (D) Transverse sections of the IVDs showing the spatial distribution of tdTomato‐positive cells in the IVDs from *Pdgfra^CreERT2^;R26^tdTomato^* mice following puncture. The dotted line indicates the AF‐NP boundary. (E) Sagittal sections of the IVDs showed spatially distributed tdTomato‐positive cells in the IVDs from *Pdgfra^CreERT2^;R26^tdTomato^* mice with and without puncture on day 14. The dotted line indicates the AF‐NP boundary. Arrowheads indicate the appearance of tdTomato‐positive cells in the NP region. (F) Quantification of the percentage of tdTomato+ cells within the innermost two layers of the AF (i.e., the two layers of AFCs near the AF‐NP boundary) at three time points. *N* = 3 per group. *p < .05 vs. control by Student's *t*‐test was considered statistically significant. Error bars represent 95% confidence intervals (CI). (G) Expression of *Pdgfra* in the rat and mouse AF on day 3 after needle puncture determined using qRT–PCR. *N* = 3 per group. **p < .05 vs. control by Student's *t*‐test was considered statistically significant. Error bars represent 95% confidence intervals (CI). (H) Immunofluorescence analysis of Ki67 in the inner layer of the AF in *Pdgfra^CreERT2^;R26^tdTomato^* mice. The dotted line indicates the AF‐NP boundary. Arrowheads indicate Ki67‐ and tdTomato‐double‐positive cells. (I) Expression levels of *Pdgfa* in each pathological region at four time points determined using stRNA‐seq data. IVD, intervertebral disc; NP, nucleus pulposus; NPC, NP cells; AF, annulus fibrosus; AFC, AF cells; stRNA‐seq, spatial transcriptomic sequencing.

Given the critical role attributed to Pdgfra+ AFCs in tissue development, as suggested by our transcriptomic analysis, we compared these cells with previously described Pdgfra+ cells implicated in NP regeneration.^58^ We found that the Pdgfra+ AFC subset identified in our study had a similar expression profile as the Pdgfra+ NPCs in a previous study.^58^ Both our Pdgfra+ AFCs and the previously reported Pdgfra+ NPCs exhibited elevated expression of *Pdgfra*, *Prrx1*, *Pla2g2a*, and *Serpinf1* (Figure 4A), indicating a shared molecular profile and close resemblance. However, in our study, the *Pdgfra* expression and spatial mapping of Pdgfra+ AFCs was primarily localized in the outer AF, but not in the NP, of the control IVD (Figures 3C and 4B). Following puncture‐induced degeneration, these cells gradually migrated toward the inner AF and appeared in the NP by day 28 (Figure 3C). Based on these findings, we hypothesized that a portion of the reported Pdgfra+ cells in the NP may originate from the AF during IVDD.

To confirm our hypothesis, we investigated the origin and descendants of Pdgfra+ AFCs and tracked their migration within the IVD using *Pdgfra^CreERT2^;R26^tdTomato^* mice. Tamoxifen was administered before needle puncture to induce red fluorescence in the progeny of Pdgfra‐expressing cells (Figure 4C). The tdTomato signal analysis (Figure 4D) revealed that Pdgfra+ AFCs were initially located in the outer AF on day 0. However, they increased in the inner AF on Days 14 and 28, indicating the migration of Pdgfra+ AFCs toward the inner AF. Notably, some tdTomato‐labeled cells were observed in the NP on day 14, confirming the migration of Pdgfra+ AFCs into the NP (Figure 4E). Quantitative analysis demonstrated a significant increase in the proportion of tdTomato+ cells within the innermost two layers of the AF following puncture compared with the control (Figure 4F). Correspondingly, qRT‐PCR revealed significant upregulation of *Pdgfra* in both the rat and mouse AF at day 3 (Figure 4G). Immunostaining of Ki67 on day 14 IVDs revealed a notable increase in AFC proliferation (Figure 4H). Notably, over 90% of the Ki67+ cells were found to be tdTomato+, suggesting active proliferation of Pdgfra+ AF lineage cells post‐puncture (Figure 4H), confirming the migration of the proliferating Pdgfra+ AF lineage cells into the inner AF and even the NP following puncture. Collectively, these results suggest that Pdgfra+ AFCs are a potential origin of the previously reported Pdgfra+ NPCs.^58^ They contribute to wound healing in response to puncture injury and may play a role in the onset of NP degeneration and fibrosis.

Pdgfra is a receptor of the platelet‐derived growth factor family. Intriguingly, we observed the highest expression of *Pdgfa* in the stress zone and NP buffer zone of the Day 1 disc (Figure 4I). These findings suggest that PDGF‐A signaling emanating from the puncture response region influences the activation of Pdgfra+ AFCs during the acute post‐puncture phase, potentially affecting their subsequent proliferation and migration.

### 3.4 Analysis of NPC clusters identified puncture‐induced Col3hi cell subsets

**FIGURE 5:**
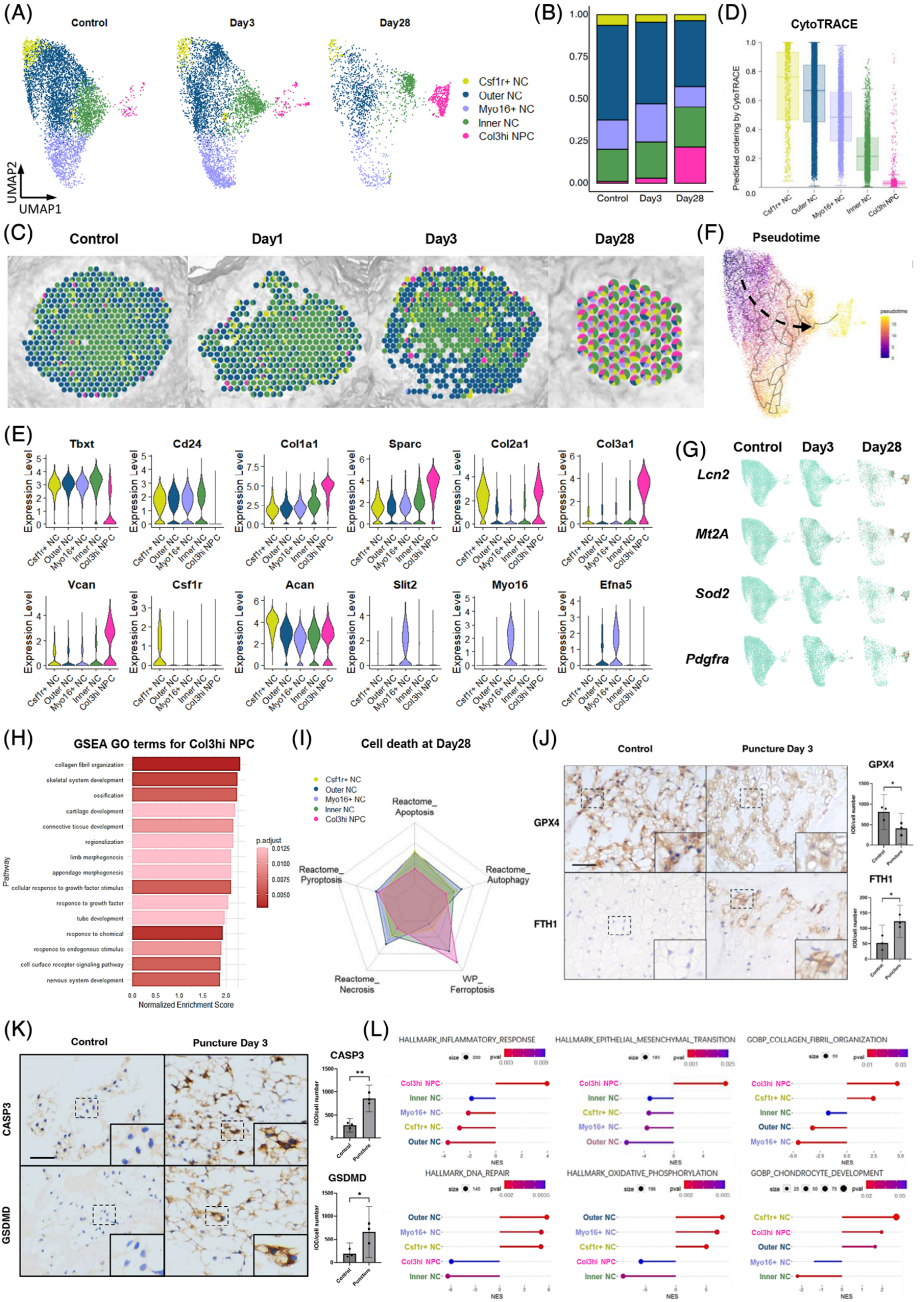
Analysis of NP cell subclusters. (A) UMAP visualization displaying the five distinct subclusters identified within the NP cell population. (B) Proportional representation of the five NPC subclusters, indicating their relative abundance. (C) Spatial distribution of the five NPC subclusters following puncture, highlighting their specific localization within the nucleus pulposus region. (D) CytoTRACE analysis predicting the developmental order of the five NPC subclusters. (E) The relative expression levels of marker genes specific to each of the five NPC subclusters. (F) Pseudotime analysis using Monocle to assess the developmental trajectory of cells. (G) Distribution and relative expression patterns of *Lcn2*, *Mt2A*, *Sod2*, and *Pdgfra* within the NPC cluster at different time points. (H) GSEA results indicating significant fibrotic and ossified changes in Col3hi NPC. (I) Radar map showing the normalized enrichment score (NES) from GSEA of cell death‐associated gene sets among each NPC subcluster at day 28 after puncture. The minimum and maximum scales correspond to NES values of .8 and 1.4, respectively. (J, K) Immunohistochemical analysis of GPX4 and FTH1 (J), Caspase 3 and GSDMD (K) in rat NP tissue from control and day 3 post‐puncture discs. Scale bar = 50 µm. *N* = 3 per group. *p < .05 vs. control by Student's *t*‐test was considered statistically significant. Error bars represent 95% confidence intervals (CI). GSDMD: Gasdermin‐D. (L) Biological process terms for all NPC subclusters obtained from GSEA analysis. NP, nucleus pulposus; NPC, NP cells; GPX4, glutathione peroxidase 4; FTH1, ferritin heavy chain; GSEA, Gene Set Enrichment Analysis.

Further re‐clustering analysis of the NPC population revealed five NPC clusters (Figure 5A). Clusters 1–4 are notochordal‐like cells (NCs), which highly expressed the notochordal markers *Tbxt* and *Cd24*. Two major NC subclusters were identified based on their spatial distribution as they did not exhibit unique marker gene expression. Inner NCs (cluster 4) were defined as those located centrally within the NP, whereas outer NCs (cluster 2) were located peripherally and adjacent to the AF. These two subclusters represent the major cellular components of these NP regions, as illustrated in Figure 5C. Csf1r+ NCs (cluster 1) were characterized by high expression of *Csf1*, a colony‐stimulating factor associated with proliferation. Myo16+ NCs (cluster 3) exhibited unique expression of *Myo16*, *Slit2*, and *Efna5*, all of which are involved in cell migration. The Col3hi NPC emerged as the only NPC cluster that largely increased following puncture (Figure 5A,B). The cluster was characterized by high *Col3a1* and reduced *Tbxt* and *Cd24* levels, solidifying their link to degeneration (Figure 5E). The spatial depiction of these NPC subsets (Figure 5C) reveals an augmentation of Col3hi NPC from the outer NP layer toward the entire region. Importantly, Col3hi NPC exhibited increased levels of *Col1a1*, *Col2a1*, *Vcan*, and *Sparc*, indicative of a fibrochondrocyte‐like phenotype and active tissue fibrosis. Consistently, the epithelial–mesenchymal transition, an important fibrosis pathway,^59^ was highly activated in Col3hi NPC (Figure 5L), indicating their potential contribution to NP fibrosis.

CytoTRACE analysis (Figure 5D) revealed that Csf1r+ NCs and Col3hi NPC exhibited the least and most differentiated subsets, respectively. Monocle pseudotime analysis using Csf1r+ NCs as the root confirmed these results and positioned Col3hi NPC at the endpoint of the differentiation trajectory (Figure 5F).

The expression of *Lcn2*, *Mt2A*, and *Sod2* was significantly increased on Day 28 NP, with a prominent enrichment in Col3hi NPC (Figure 5G). GO analysis of the Col3hi NPC (Figure 5H) revealed a pronounced synthesis of collagen fibrils, indicative of a phenotype resembling chondrocyte‐like cells. Importantly, we found increased *Pdgfra* expression in degenerated Col3hi NPCs on Day 28 after puncture (Figure 5G), suggesting another crucial source of *Pdgfra+* NPCs. Collectively, these findings indicate that *Pdgfra+* cells within the degenerated NP exhibit a dual origin, deriving from both the AF (Pdgfra+ AFC) and NP (Col3hi NPC) lineages.

Cell interaction analysis (Figure S4) revealed that Pdgfra+ AFCs are regulated by Myo16+ NCs and Inner NCs through PDGF signaling. Multiple subgroups of AFCs influenced Outer NCs and Myo16+ NCs through the bone morphogenetic protein signaling pathway, thereby playing a role in the ossification process of these cells. Myo16+ and Outer NCs may affect Lcn2+ AFCs through pleiotrophin signaling. In contrast, Lcn2+ AFCs may stimulate Csf1r+ NCs through secreted phosphoprotein 1 signaling.

### 3.5 Cell death analysis revealed that ferroptosis contributes significantly to NPC loss

Since NPCs were the only category that decreased post‐puncture, to understand the cause of NPC loss, we assessed various cell death processes by GSEA at 28 days post‐puncture (Figure 5I). Ferroptosis was observed in Col3hi NPC and Inner NCs, whereas other cell death modes were minimally detected. We further examined key markers of ferroptosis, pyroptosis, necrosis, and apoptosis in NPCs (Figure S5A). Ferroptosis was induced as early as day 3 post‐puncture, with a further increase by day 28. In contrast, other types of cell death were observed at moderate to low levels. Analysis of gene sets from the molecular signatures database (MSigDB) corroborated these findings, demonstrating a high level of ferroptosis and minimal pyroptosis, necrosis, and apoptosis of NPCs (Figure S5B). Furthermore, immunohistochemical analysis of rat NP tissue revealed reduced expression of the ferroptosis inhibitor glutathione peroxidase 4 (GPX4) and increased expression of ferritin heavy chain 1 (FTH1) at 3 days post‐puncture (Figure 5J). The reduction in GPX4, a key regulator of ferroptosis,^60^ and the increase in FTH1, an indicator of iron accumulation associated with ferroptosis,^61^, ^62^ strongly suggest the early induction of ferroptosis in the NP following puncture (Figure 5J; Figure S5A).

Besides ferroptosis, the roles of apoptosis and pyroptosis in puncture‐induced IVDD have been widely studied and are considered important contributors to the degenerative process.^63^ We further compared the expression of Caspase‐3, a marker for apoptosis, and GSDMD, a marker for pyroptosis, between puncture‐injured NP tissue and control samples. Our results showed that both Caspase‐3 and GSDMD expression levels were increased following puncture injury (Figure 5K). Combined with the broader transcriptomic data from our snRNA‐seq analysis (Figure S5B), these findings indicate that apoptosis and pyroptosis pathways were also induced after puncture injury.

### 3.6 stRNA‐seq revealed distinct puncture response regions at the puncture site

**FIGURE 6:**
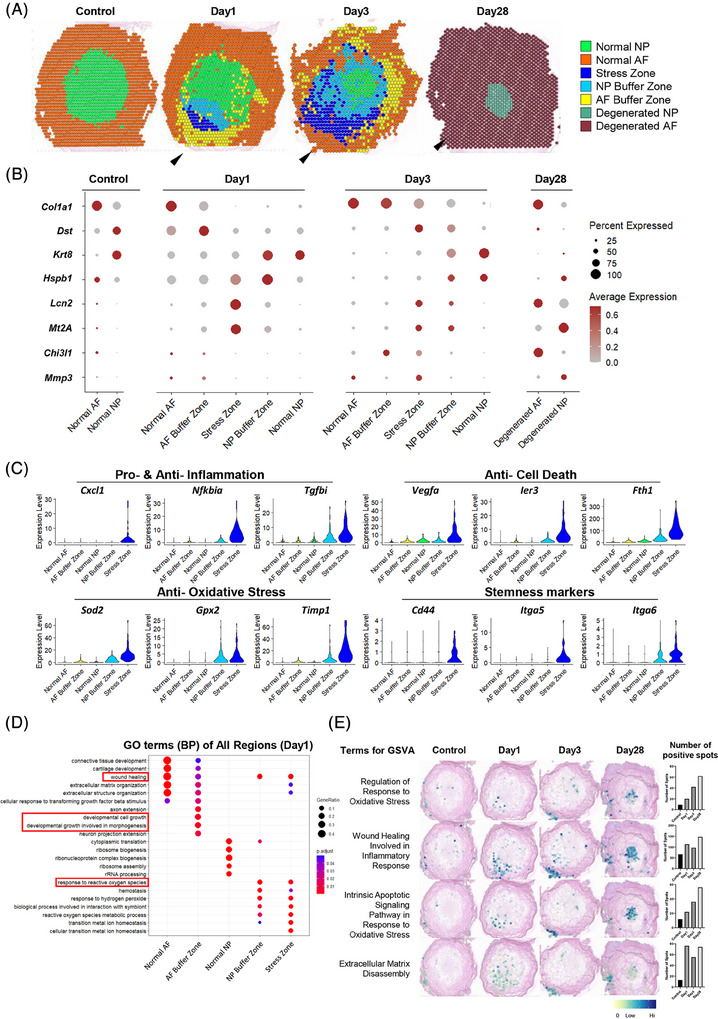
Puncture response regions identified in the transcriptomic analysis of the IVD puncture rat model. (A) Spatial distribution of pathological regions within the IVD at different time points following puncture. Arrowheads indicate the direction of the puncture. (B) Expression levels of marker genes specific to each identified zone within the IVD. (C) Marker genes associated with the main biological processes occurring in the stress zone. (D) GO analysis results showing the biological process terms associated with all regions on day 1. (E) Spatial gene set variation analysis results showing the variation in gene sets across all time points. IVD, intervertebral disc; GO, gene ontology.

In addition to the cell subsets, we further investigated the spatial area in response to puncture by stRNA‐seq. Distinct zones were defined using a combined approach of clustering analysis and histological referencing (Figure 6A). The annulus fibrosus (AF) and NP in the unpunctured control IVD were defined as normal AF and normal NP respectively, characterized by the expression of the healthy‐specific markers *Col1a1* and *Krt8* (Figure 6B). By day 28, a decrease in *Krt8* expression within the NP indicated phenotypic integrity loss, while the increased concurrent expression of the degenerative marker *Chi3l1* ^64^, ^65^ in the AF and *Mmp3* ^66^, ^67^, ^68^ in the NP further corroborated this degenerative process, enabling identification of Degenerated AF and Degenerated NP zone, respectively (Figure 6B).

Notably, a distinctive region emerged at the puncture site (arrowheads in Figure 6) on both days 1 and 3. An area, termed as “Stress Zone”, exhibited elevated expression of oxidative stress‐related genes (*Lcn2*, *Mt2A*, and *Sod2* ^69^, ^70^, ^71^), emphasizing oxidative stress as the primary pathway (Figure 6B). Differential gene expression analysis further revealed upregulation of inflammation‐related genes (*Cxcl1*,^72^ *Nfkbia*,^73^ and *Tgfbi* ^74^), antiapoptotic and antiferroptotic genes (*Vegfa*; ^75^ *Ier3*; ^76^ *Fth1* ^77^), and stemness markers (*Cd44*,^78^ *Itga5*,^79^ and *Itga6* ^80^), suggesting the initiation of complex biological changes in this localized region (Figure 6C).

Additionally, two zones were identified to connect the Stress Zone and the AF/NP regions (arrowheads in Figure 6A). These zones maintained the phenotype of the original tissues that expressed AF/NP markers (*Col1a1* and *Krt8*). Several pathology‐related genes were significantly upregulated in these regions (Figure 6B), including *Dst* (encoding dystonin, crucial for maintaining cytoskeletal integrity^81^, ^82^), *Hspb1* (encoding heat shock protein B1, which protects cells against oxidative stress^83^, ^84^), and *Chi3l1* (encoding chitinase‐3 like‐protein‐1, involved in tissue injury and repair^85^). Gene ontology (GO) analysis revealed characteristics related to wound healing and cell growth, suggesting that they function as buffer areas with regenerative processes against stress‐related damage (Figure 6D). Consequently, we designated these areas as the AF buffer zone and NP buffer zone, respectively. Collectively, these two zones and the stress zone constitute the puncture response region, which expanded from days 1 to 3. GSVA analysis further revealed a progressive increase in the occurrence of oxidative stress and associated apoptosis, while inflammatory healing and matrix degradation persisted at elevated levels after puncture (Figure 6E). These findings highlight the complex spatial dynamics within the injured IVD and underscore the critical roles of the above pathological processes in these functional zones in transforming acute injury into degenerative changes.

**FIGURE 7:**
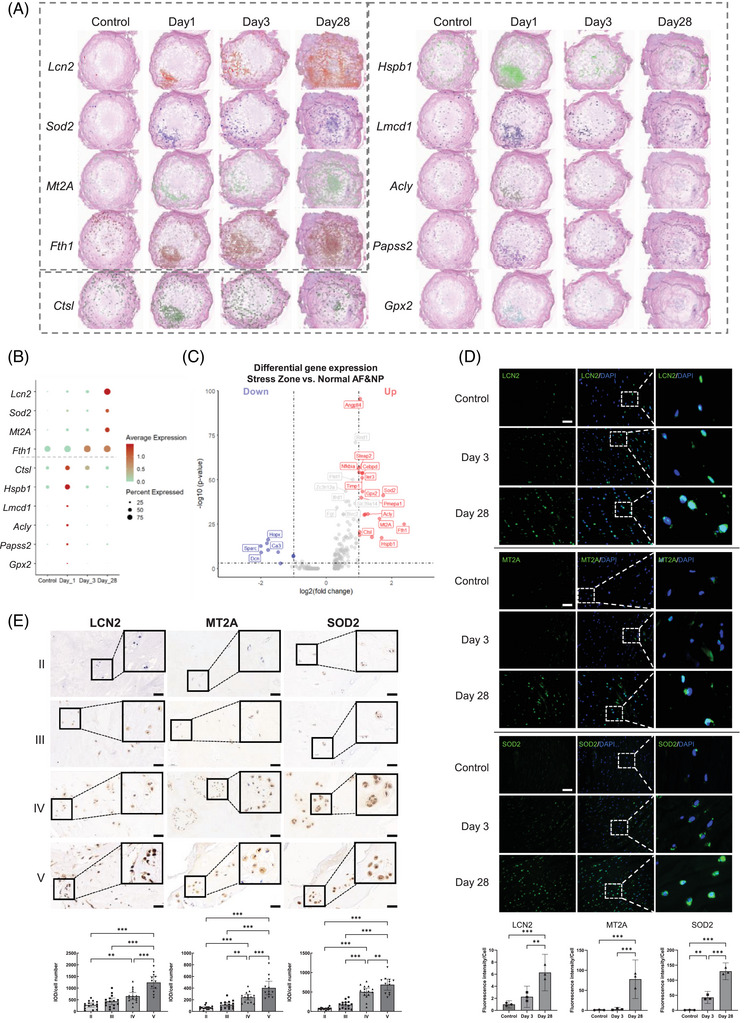
Top DEGs in the stress zone after puncture were associated with oxidative stress. (A) Spatiotemporal distribution of upregulated genes observed in the stress zone. (B) Expression levels of upregulated genes observed in the whole IVD at different time points, showing two expression patterns. (C) Differential gene expression analysis comparing the puncture response region to the normal region. (D) Representative immunofluorescence staining of LCN2, MT2A, and SOD2 in the inner AF region of the rat IVD puncture model. Scale bar: 50 µm, *N* = 3 per group. **p < .01 and ***p < .001 by one‐way ANOVA with post hoc comparisons were considered statistically significant. Error bars represent 95% confidence intervals (CIs). (A) Immunohistochemical analysis of LCN2, MT2A, and SOD2 expression in human IVD tissues according to Pfirrmann grade. Scale bar: 50 µm. **p < .01 and ***p < .001 by one‐way ANOVA with post hoc comparisons were considered statistically significant. Error bars represent 95% confidence intervals (CIs). The sample size per group and demographic data of IVD donors are detailed in Table S2. DEG, differential expressed genes; IVD, intervertebral disc; AF, annulus fibrosus; LCN2, lipocalin‐2; MT2A, metallothionein‐2; SOD2, superoxide dismutase 2.

We conducted differential gene expression (DEG) analysis comparing the stress zone to both normal AF and NP regions on day 1. The complete list of DEGs is provided in Table S1. The temporal dynamics of spatial distribution and expression levels for the top 10 DEGs are illustrated in Figure 7A. Notably, Lcn2, Mt2A, Sod2 (superoxide dismutase 2),^86^ and Fth1 continuously increased in the whole IVD to day 28 (Figure 7B), while the other six exhibited temporary upregulation on day 1 but decreased afterward till day 28. Lcn2, Mt2A, Sod2, and Fth1 are closely indicated in oxidative stress and ferroptosis. Lcn2+ AFCs are the predominant cell component within the stress zone (Figure 3C). Hence, we selected Lcn2, Mt2A, and Sod2 for further validation. Immunostaining in punctured rat discs (Figure 7D) and human NP specimens with increasing IVDD severity confirmed the upregulation of LCN2, MT2A, and SOD2 (Figure 7E) in IVDD. Meanwhile, ferritin heavy chain (FTH1)^77^ and glutathione peroxidase 2 (GPX2)^87^ are closely associated with ferroptosis. These findings indicate that oxidative stress and ferroptosis are the primary responses to puncture, suggesting their potential role in triggering degeneration.

## 4 DISCUSSION

By employing stRNA‐seq and snRNA‐seq, we revealed oxidative stress as a prominent pathway in an immediate puncture response region termed “Stress Zone” on day 1 post‐puncture. Interestingly, two distinct categories were identified among acute response genes around the stress zone (Figures 7A and 6B). The first category (*Lcn2*, *Sod2*, *Mt2A*, and *Fth1*) was significantly induced at the puncture response region on day 1 and continuously increased till day 28. The second category (*Ctsl*, *Hspb1*, *Lmcd1*, *Acly*, *Papss2*, *Gpx2*) exhibited temporary upregulation on day 1 but gradually decreased till day 28. The underlying reasons for the distinct behaviors of these two categories of genes remain unclear and warrant further investigation.

We identified several IVD cell subclusters induced during degeneration, including Lcn2+ AFC, Col3hi NPCs, and Pdgfra+ AFCs. Our findings highlight a significant role of LCN2+ AFCs as the primary cell type within the Stress Zone. LCN2 has both pro‐oxidant and antioxidant^54^ properties, as well as the ability to induce ferroptosis and inflammation.^55^ Increased LCN2 expression has been observed in IVD cells in response to nerve growth factor‐induced degeneration.^66^, ^88^ Here, we demonstrated a significant correlation between LCN2 expression and degeneration in human and rat IVDs. Elevated expression of *Sod2* and *Mt2A* indicated enhanced antioxidant activity within Lcn2+ AFCs.^56^, ^86^ Furthermore, Lcn2+ AFCs demonstrated high expression of chemokines, such as *Cxcl14*, and complement factors, including C3 and C1, which play crucial roles in initiating immune response processes. Collectively, these findings indicate that Lcn2+ AFCs play a pivotal role in connecting oxidative stress with other pathological processes involved in IVD degeneration within the stress zone.

Since our sequencing data were obtained from rats, to understand whether our findings are applicable to humans, we look into human scRNA‐seq datasets on human IVDs in the literature. Han et al.^89^ performed sc‐RNA seq of human NP and found that chondrocytes in the NP can be defined into three populations, including fibrochondrocyte progenitors, which may have a close resemblance with our Col3hi NPC. Both Han's study^89^ and Ling's study^90^ identified macrophages in the human NP, which is consistent with our findings. Gan's study^58^ uncovered PDGF cascade as important in the NP microenvironment, which confirmed the importance of PDGF signaling in our work. Our findings support recent evidence of NP cell turnover from the outer to the inner region, as evidenced by the distinctions observed in the NP subclusters between the outer and inner NP layers.^91^

Moreover, our spatial transcriptomics and lineage tracing allowed us to identify that the Pdgfra+ NP cells in another study^58^ may be partially derived from Pdgfra+ AFCs. They migrate from the outer AF toward the NP during degeneration, which may explain why this population was first identified as NPPC in the other study. Further intravital cell imaging will help to visualize the migration patterns of Pdgfra+ AFCs and the dynamics of the stress zone. We also observed the highest expression of *Pdgfa* (encoding a key ligand for PDGFR) in the stress zone and NP buffer zones of Day 1 discs (Figure 4I). Cell interaction analysis revealed the receipt of PDGF signals from multiple cell types to Pdgfra+ AFCs, indicating PDGF as the upstream stimulator of Pdgfra+ AFCs. These findings suggest that PDGF‐A signaling originating from the puncture response region influences the activation of Pdgfra+ AFCs during the acute post‐puncture phase. However, the exact mechanism by which the IVDD microenvironment leads to the migration and differentiation of Pdgfra+ AFCs awaits further study.^92^, ^93^

However, Pdgfra+ AFC is not the only source of the *Pdgfra+* cells in the degenerated NP. Importantly, the puncture‐induced Col3hi NPC also has increased *Pdgfra* expression compared with the other NP cell subpopulations at day 28 after the puncture (Figure 5G), suggesting another crucial source of *Pdgfra+* NP cells. These findings collectively indicated that the increased *Pdgfra* expression in the degenerated NP exhibits a dual origin, deriving from both Pdgfra+ AFC and Col3hi NPC. It is likely that Pdgfra+ AFCs migrating from the AF may contribute to the initial fibrotic response, while Pdgfra+ Col3hi NP cells may contribute to the later stages of fibrosis as the NP matrix deteriorates. However, the lineage tracing data are mouse‐derived, and further research in rats and humans is needed to fully understand Pdgfra+ cell origins and roles across species, as well as the precise temporal dynamics and functional contributions to NP fibrosis.

We previously reported the onset of fibrotic changes in IVDD,^12^, ^13^ but the cause of fibrosis in the degenerated NP currently remains in debate. Some demonstrated fibrosis of NPC during IVDD, while some others argue that the increase in fibrotic markers in the NP may be due to AFC migrating into the NP after AF rupture and degeneration, resulting in increased stiffness in the NP, since AFC have higher mechanical properties than NPC. In our study, we clarified that there was indeed a migration of Pdgfra+ AFC into the NP post‐puncture, highlighting the contribution of AF cells to NP fibrosis since AFC has higher mechanical stiffness than NPC. On the other hand, the emergence of the Col3hi NPC subpopulation which expressed high fibrosis‐related markers and EMT activation post‐puncture also indicated fibrotic transition of NP cells themselves during degeneration. Therefore, Col3hi NPC and Pdgfra+ AFC may both contribute to NP fibrosis via distinct mechanisms, highlighting the complexity of the process. While our data clarify their origins and roles, future functional studies would be needed for direct comparative quantification of their fibrogenic potential.

The identification of oxidative stress markers such as LCN2 and Mt2A in the initiation and deterioration of IVDD indicates their potential as markers of IVDD. The identification of Lcn2+ AFCs as key initiators of the stress response in the “Stress Zone” suggests that targeting Lcn2+ AFCs or the pathways they activate (such as oxidative stress and ferroptosis) holds potential therapeutic relevance for interrupting the early cascade of IVDD. We also identified Pdgfra+ AFCs which exhibit progenitor potential and migrate from the AF into the NP following puncture. While potentially involved in wound healing, our findings suggest they may also contribute to NP degeneration and fibrosis. Targeting Pdgfra signaling, which influences the activation of Pdgfra+ AFCs, could offer a strategy to modulate their migration, proliferation, or differentiation, thereby potentially mitigating fibrotic changes in the degenerating disc. While our snRNA‐seq and stRNA‐seq data provide strong correlative evidence of their spatial and temporal dynamics, functional interactions require further study. Future works employing knockdown/knockout experiments for deeper mechanistic exploration are desired to reveal their role in IVD degeneration and fibrosis.

Our study identifies Lcn2+ AFCs as key initiators of the stress response in the “Stress Zone” immediately post‐puncture. These cells play a pivotal role in linking oxidative stress to downstream pathological processes, including ferroptosis, fibrosis, and immune reactions. We also identified Pdgfra+ AFCs which exhibit progenitor potential and migrate from the AF into the NP following puncture. While potentially involved in wound healing, our findings suggest they may also contribute to NP degeneration and fibrosis. Therefore, further knockdown/knockout experiments, co‐culture assays, conditional lineage tracing, or single‐cell secretome analysis may facilitate understanding the specific roles of Lcn2+ AFCs and Pdgfra+ AFCs in initiating and propagating key pathological processes in IVDD and opens new avenues for targeted therapeutic interventions aimed at preventing or slowing down disc degeneration. The detection of ferroptosis in IVDD suggests a possible clinical strategy to target IVDD through inhibiting ferroptosis. Further investigation of the role of the pathways highlighted in IVDD, including PDGF, BMP, complement, and IL16 signaling, would help to disclose the cross‐talk of cell‐cell interactions in the degeneration process. Taken together, these advances may hold potential for future therapeutic prevention or mitigation of IVD degeneration.

Our snRNA‐seq data show a relatively higher number of detected genes (features) and lower UMI counts (nCounts) per nucleus compared with typical expectations for scRNA‐seq. However, this pattern is, in fact, consistent with the expected differences between the two technologies. snRNA‐seq captures nuclear RNA (including pre‐mRNA), leading to the detection of more diverse transcripts (higher features), while scRNA‐seq captures whole‐cell RNA (including abundant cytoplasmic mRNA), resulting in higher total UMI counts.^94^, ^95^ Our snRNA‐seq data, with median genes per cell ranging from 1818 to 3236 and median UMI counts per cell ranging from 1113 to 1604, aligns with these expected patterns for nuclear transcriptomes and indicates good data quality. Another primary factor contributing to this discrepancy is likely the enzymatic dissociation used in standard scRNA‐seq. This process can induce stress responses and other artifacts, altering gene expression profiles, particularly for sensitive transcripts. Our snRNA‐seq approach, however, analyzes frozen nuclei without enzymatic digestion, better preserving the in vivo transcriptional state.^50^, ^51^

We recognize that our use of the rodent IVD puncture model, while primarily replicating annular injury‐induced IVDD and being suitable for studying IVD cellular responses to injury, does not fully encompass all facets of human IVDD, including endplate damage, genetic influences, and cellular composition. The use of transverse sections, implemented to optimize RNA integrity by precluding decalcification procedures,^24^ resulted in the exclusion of the analysis of cartilage endplate (CEP) in our study, which is also important to understand the mechanism of IVDD. Future studies examining CEP cell spatial dynamics in IVDD will necessitate modification of decalcification protocols to maintain RNA integrity. In addition, human IVD starts to degenerate from 10 years of age, when notochordal‐like cells start to disappear in the NP.^96^, ^97^ Some recent studies have also identified notochordal‐like cells in adult human NP.^58^, ^98^, ^99^ It is possible that the presence of abundant notochordal‐like cells in rodent NP influences the degenerative process differently than in humans. However, our study also identifies conserved mechanisms of IVD degeneration across species. These include key processes such as oxidative stress, AF cell migration, and the involvement of fibrosis‐related NP cells, all of which are relevant to human IVDD. Validation of these conserved findings using human IVD samples strengthens their clinical relevance. Therefore, our study may more resemble the very early stage of human IVD degeneration and suggest translational potential for clinical applications by targeting these conserved mechanisms of IVD degeneration observed across species. Besides, the snRNA‐seq lacks data for day 1, limiting insight into immediate nuclear transcriptomic changes post‐puncture. Our day‐1 spatial information complements the cellular resolution of snRNA‐seq at day 3, providing a strong approach to capture the cellular spatiotemporal dynamics of IVDD. However, our study revealed core degenerative mechanisms in these cells, such as oxidative stress and ferroptosis, which are generally conserved across species. Our analysis of human NP samples also supported the relevance of these mechanisms in human degeneration. Further studies directly comparing rodent and human IVD degeneration are necessary to fully understand the impact of this species difference.

## 5 CONCLUSION

Puncture‐induced oxidative stress plays an important role in initiating IVDD. Several puncture‐induced cell subpopulations were identified, including Lcn2+ AFC, Col3hi NPCs, and Pdgfra+ AFCs. Puncture induced the appearance of Lcn2+ AFCs in a puncture response region ‘Stress Zone’ which migrated into the NP during degeneration, playing a key role in transforming injury signaling to multiple cell types and resulting in other pathological processes, including ferroptosis, fibrosis, and immune reactions. Puncture also induced fibrochondrocyte‐like Col3hi NPCs, and stimulated Pdgfra+ AFCs proliferating and migrating from the outer AF to NP, both of which may contribute to NP fibrosis, for which further investigation is warranted.

## AUTHOR CONTRIBUTIONS

Sequencing and initial data processing were performed by Shanghai OE Biotechnology Company Limited and Guangzhou Genedenovo Biotechnology Company Limited. Subsequent bioinformatic analyses of the spatial transcriptomic and snRNA‐seq data, including data integration, dimensionality reduction, clustering, cell type annotation, and deconvolution, were performed by Guoyan Liang, Jing Tan, and Feng‐Juan Lyu. Guoyan Liang analyzed the transcriptomic data, performed lineage tracing, and drafted the paper. Jing Tan obtained sequencing data and immunostaining data and drafted the paper. Chong Chen obtained human IVD samples. Yuying Liu obtained immunostaining data. Yongyu Ye obtained human NP samples. Qiujian Zheng provided support and resources. Xiaolin Pan obtained qRT‐PCR data. Yunbing Chang supervised the study, provided support, and collected human IVD samples. Feng‐Juan Lyu designed and supervised the study, analyzed the data, and drafted the paper. All authors approved the final manuscript.

## CONFLICT OF INTEREST STATEMENT

The authors declare no conflict of interest.

## ETHICS STATEMENT

All procedures were performed in compliance with relevant laws and institutional guidelines and have been approved by the appropriate institutional committees. Ethical approval for animal experiments was obtained from the Ethics Committee of the Medical School of South China University of Technology (AEC2019040, 2019). The collection of human NP specimens was approved by the institutional review board of the hospital, and written informed consent were obtained from all patients (KY‐Z‐2020‐612‐03, 2021). Experiments involving human samples were approved by the Ethics Committee of Guangdong Provincial People's Hospital (No. KY2023‐629‐01). Informed consent was obtained from all patients before collecting IVD samples during surgery.

## Supporting information



Supporting Information

Supporting Information

Supporting Information

## Data Availability

The raw sequencing data generated in this study are available from the authors upon reasonable request. The detailed demographic information of the donors of human IVD samples, including age, gender, BMI, surgical type, surgical level, were provided in the Supporting Information Table S2.
