# Supplementary material for: Single nuclear‐spatial transcriptomic sequencing reveals distinct puncture‐induced cell subpopulations in the intervertebral disc of a rat model
Source: Clin Transl Med. 2025 Jun 13;15(6):e70370. doi: 10.1002/ctm2.70370 (PMC12166128; doi:10.1002/ctm2.70370)
Supplement: Supplementary file 1 — Supporting Information [file CTM2-15-e70370-s001.docx]

**Supplementary Figures**

**
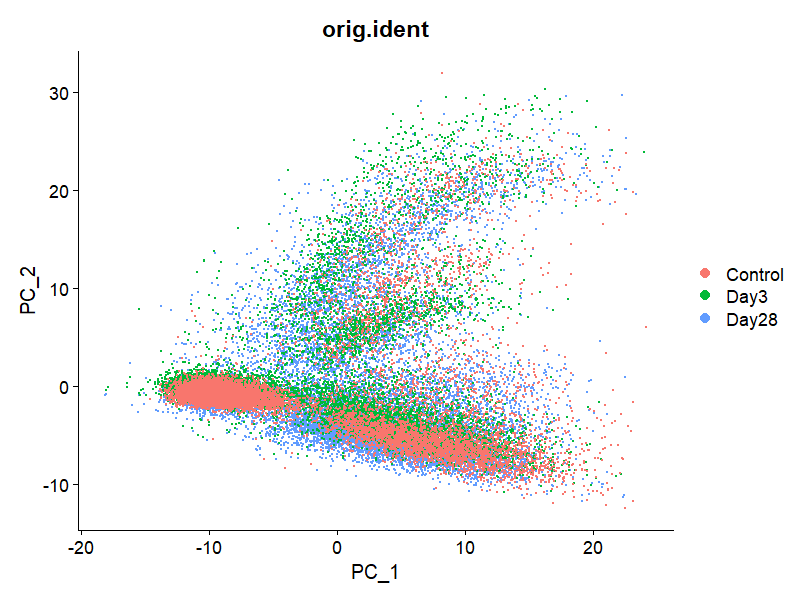
**

**Figure S1: Principal component analysis (PCA) plot illustrating the distribution of snRNA sequences from rat IVDs.**


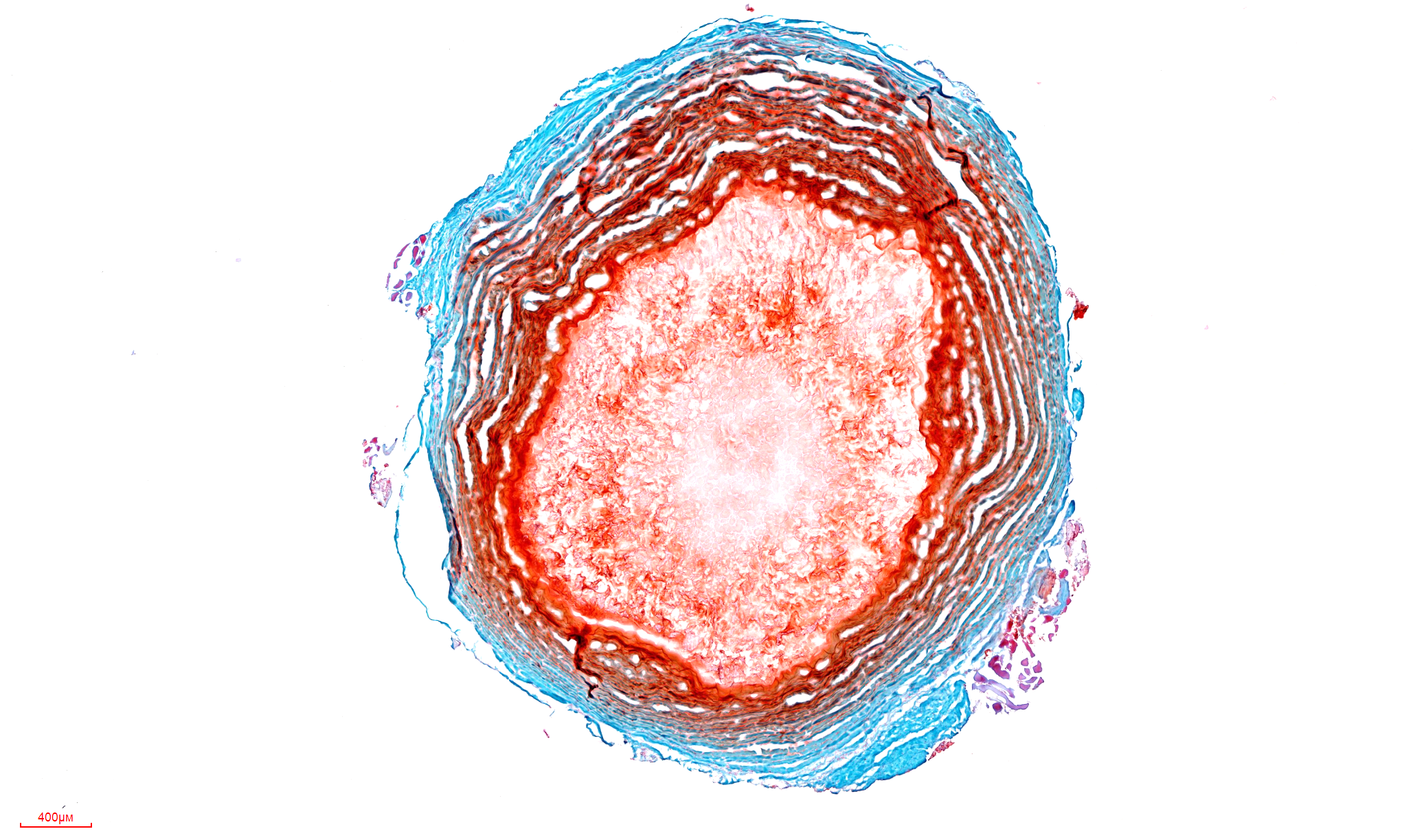

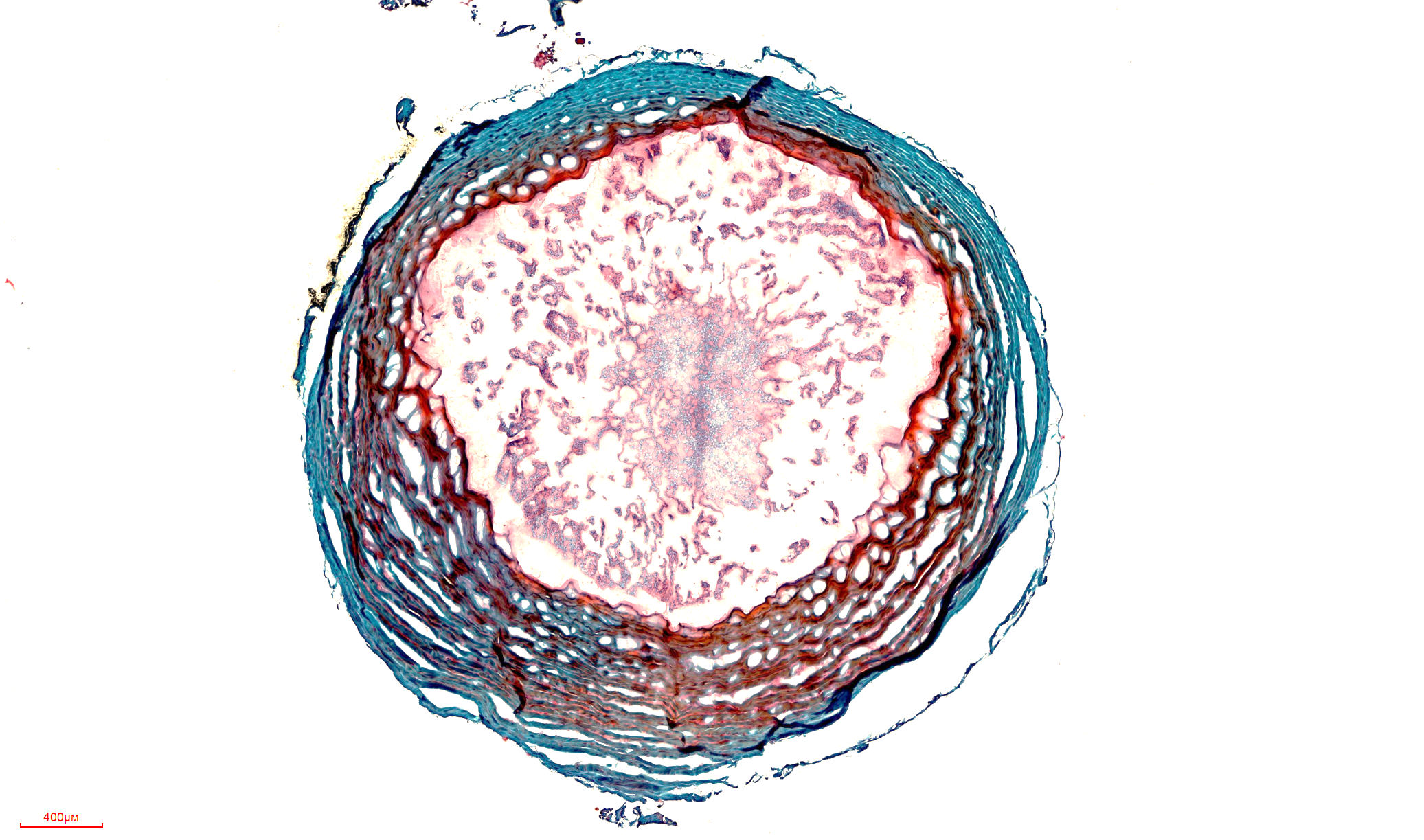

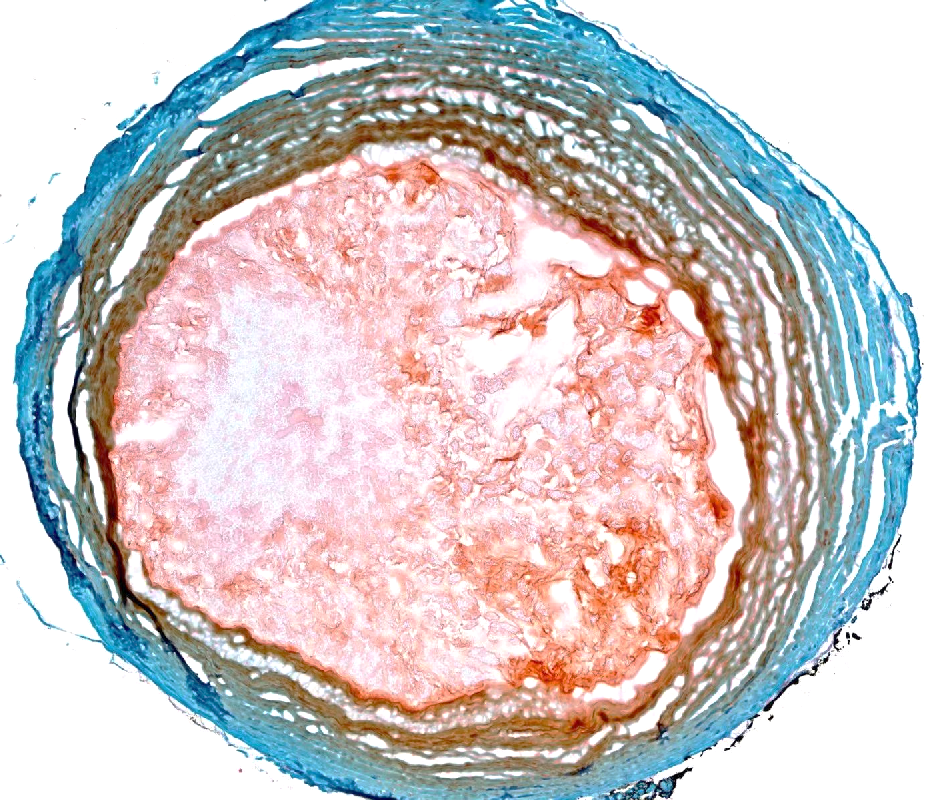


**Control**

**Day 1**

**Day 3**

**Day 28**


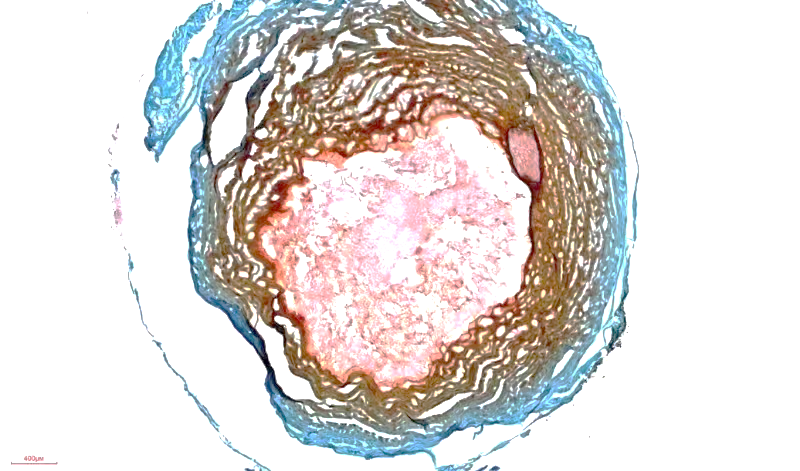

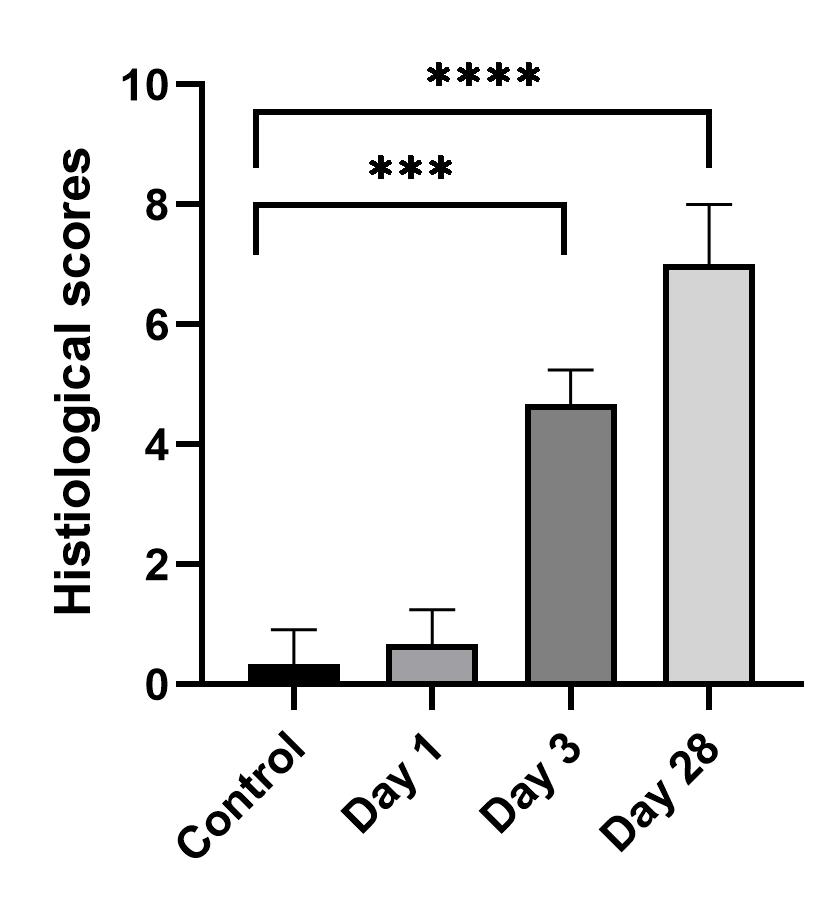


**Figure S2. Safranin O / Fast Green staining of rat intervertebral disc.** Bar graph depicts histological scores of IVDs from the four experimental groups. ****P*<0.001, ****P*<0.0001.


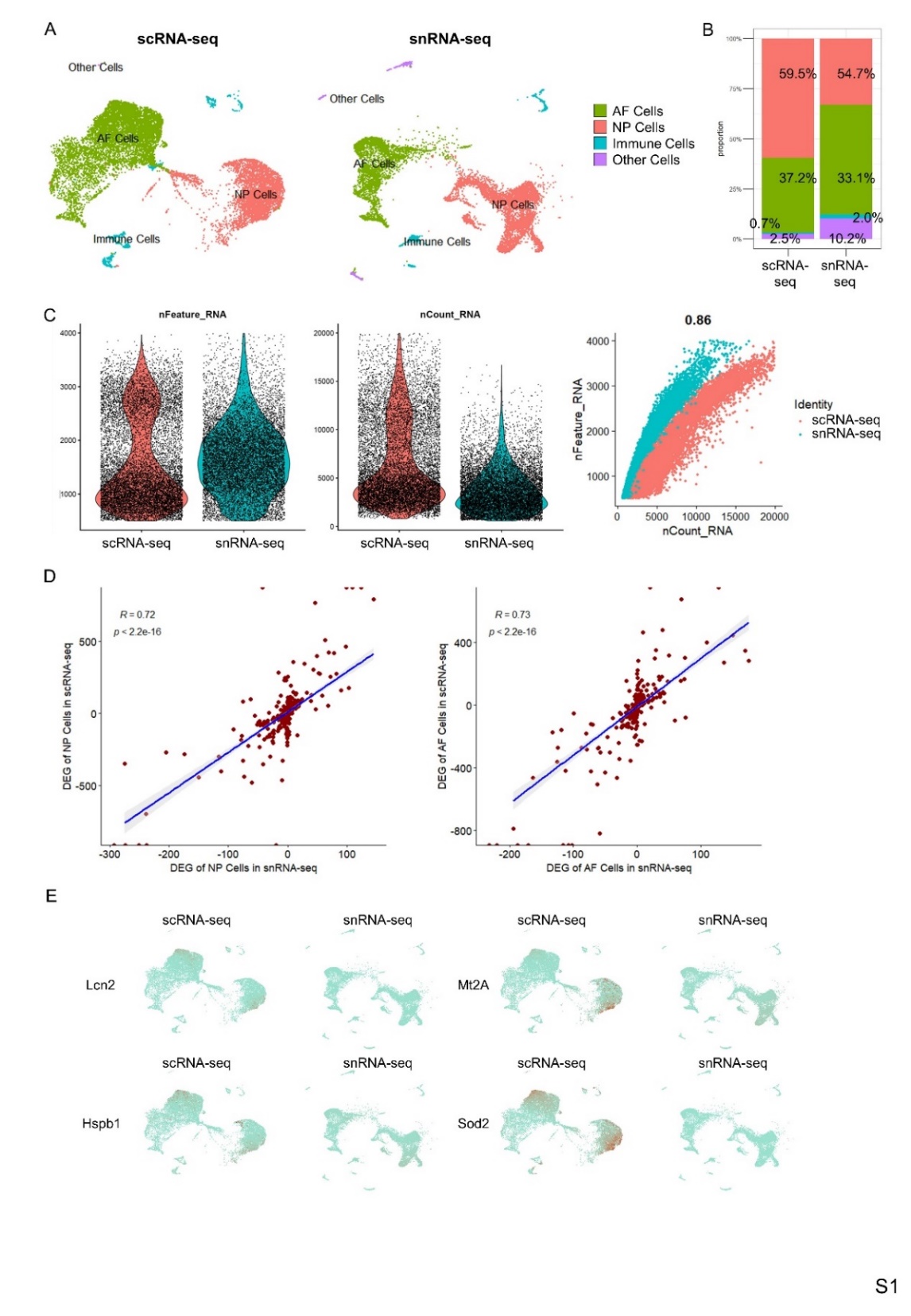


**Figure S3. Comparison of scRNA- and snRNA-seq methods revealed differences in stress-responsive gene detection.** A. UMAP visualization of rat IVD cells obtained through scRNA-seq (GSE154884) and snRNA-seq methods. B. The scRNA-seq method detected a higher proportion of AF cells (37.2% vs. 33.1%) and NP cells (59.5% vs. 54.7%), whereas the snRNA-seq method identified a higher proportion of immune cells and other cell types. C. Comparison of features and count data processed by the two methods, including the number of genes detected per cell (nFeature) and the sum of expression levels of all genes detected in each cell (nCount). Despite the lower total detection abundance in snRNA-seq, the total counts were similar between the two methods. D. Correlation analysis demonstrating the differentially expressed genes (DEGs) in NP and AF identified by both methods showed good consistency (NP: R=0.72, AF: R=0.73). E. UMAP plots displaying the differential expression of oxidative stress-related genes between the two methods.


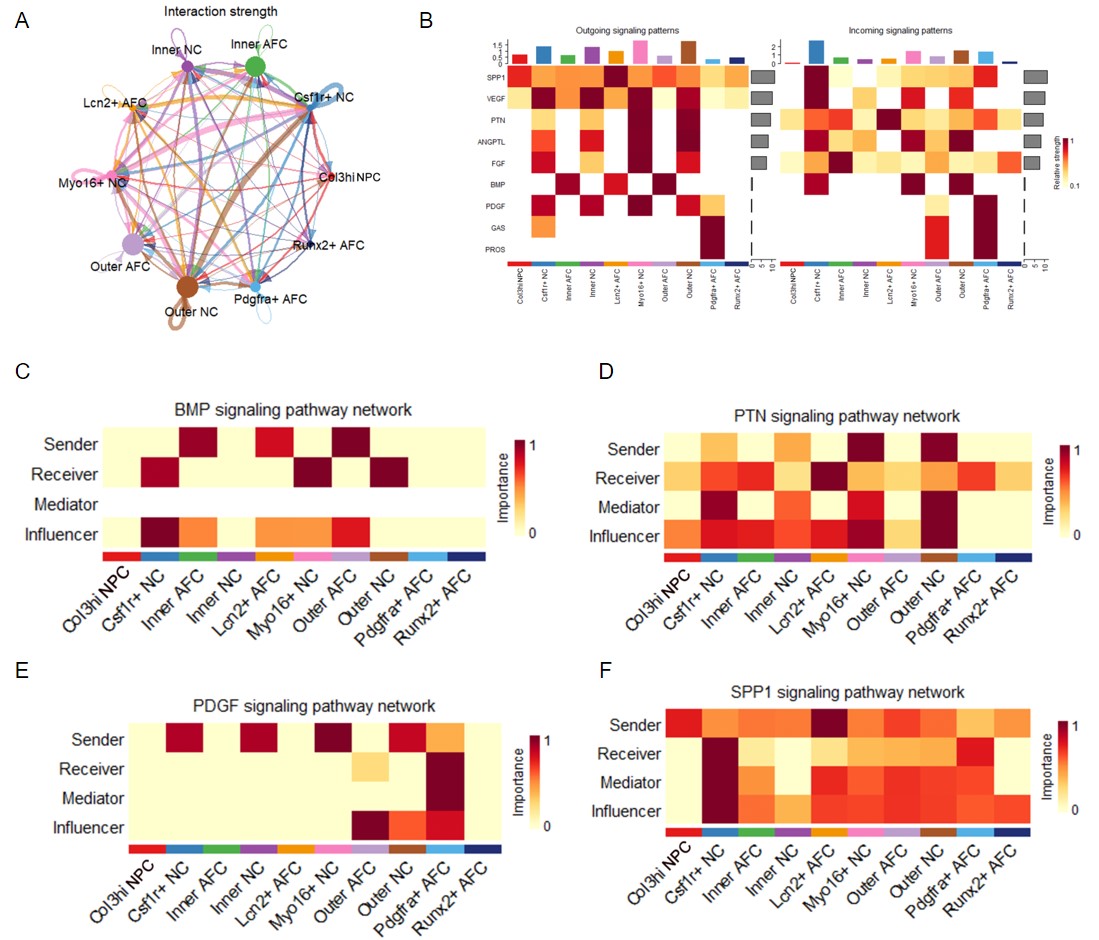


**Figure S4. Cell-cell interaction between subgroups from AF and NP cells.** A. Cell-cell interaction between AFC and NPC. B. Outgoing and incoming patterns of different signaling pathways between AFC and NPC. C. BMP signaling network between AFC and NPC. D. PTN signaling network between AFC and NPC. E. PDGF signaling network between AFC and NPC. F. SPP1 signaling network between AFC and NPC.


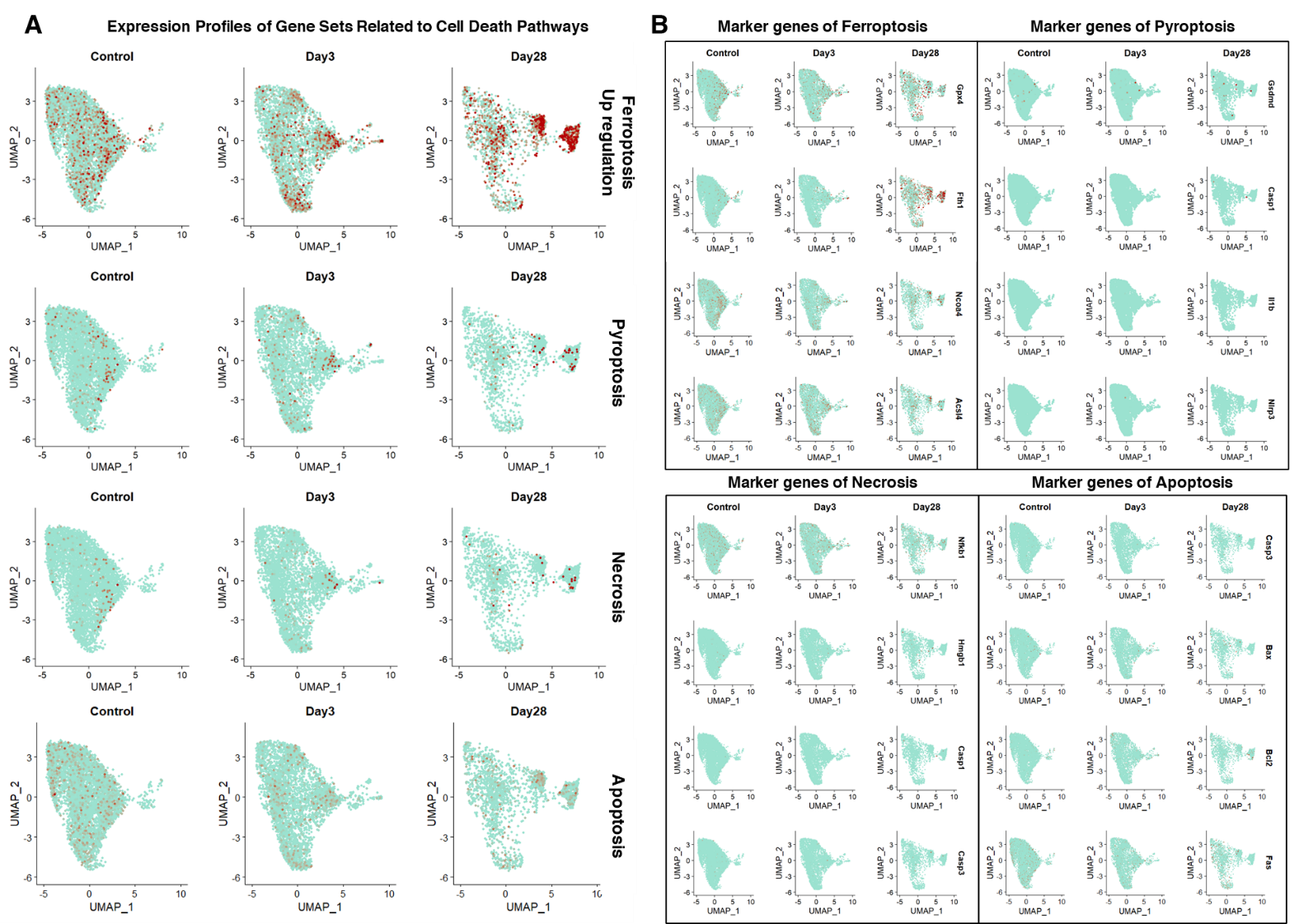


**Figure S5. Evaluation of ferroptosis, pyroptosis, necrosis, and apoptosis in NP cells after disc puncture in rats.** A. Feature plots depicting the expression of gene sets associated with ferroptosis, pyroptosis, necrosis, and apoptosis in NP cells from rat punctured discs. (B) Expression levels of gene sets from the Molecular Signatures Database (MsigDB, https://www.gsea-msigdb.org/gsea/msigdb/mouse/collections.jsp) for ferroptosis, pyroptosis, necrosis, and apoptosis.
